# Platelets induce epithelial to mesenchymal transition in renal proximal tubular epithelial cells through TGF-β signaling pathway

**DOI:** 10.1186/s10020-025-01355-7

**Published:** 2025-10-29

**Authors:** Ukhti Jamil Rustiasari, Melissa Uil, Xiaomeng Zhang, Nike Claessen, Loes Butter, Sandrine Florquin, Alessandra Tammaro, Joris J.T.H. Roelofs

**Affiliations:** 1 Department of Pathology, Amsterdam UMC location University of Amsterdam, Amsterdam, The Netherlands; 2 Amsterdam Cardiovascular Sciences, Diabetes and Metabolism, Amsterdam, The Netherlands; 3 Department of Anatomical Pathology, Faculty of Medicine, Universitas Islam Indonesia, Yogyakarta, Indonesia; 4 Amsterdam Infection & Immunity, Amsterdam, 1105AZ The Netherlands

**Keywords:** Chronic kidney disease, Fibrosis, Epithelial-mesenchymal transition, Platelets, TGF-β1

## Abstract

**Background:**

Management of chronic kidney disease (CKD) remains a major challenge due limited therapeutic options to reverse fibrosis, which is a critical feature in CKD. Partial epithelial-to-mesenchymal transition (EMT) of tubular epithelial cells (TECs) is a key driver of fibrosis, and has become an important focus for kidney protection strategies. Blood platelets, a major source of circulating transforming growth factor beta (TGF-β), are implicated in pathogenesis of CKD, but their involvement in EMT and kidney fibrosis remains uncertain.

**Methods:**

We used two mouse models of renal fibrosis—diabetic kidney disease (DKD) and unilateral ureter obstruction (UUO)—to examine the connection between platelets, partial EMT, and fibrosis. Platelet inhibition or depletion was performed to assess EMT, cell cycle arrest, and fibrosis. In vitro, platelets were applied to TECs and kidney organoids. To determine the role of TGF-β signaling, we used TGF-βRI inhibitor. Expression of EMT, and fibrosis markers, as well as TGF-β1 signaling, were analyzed using western blot, reverse transcription quantitative PCR (RT-qPCR), enzyme-linked immunosorbent assay (ELISA), and immunostaining.

**Results:**

In both animal models, platelet inhibition or depletion resulted in reduced expression of cell cycle arrest marker p21, partial EMT and fibrosis. In vitro, activated platelets stimulated cell cycle arrest, EMT, and fibrosis in TECs and kidney organoids. Chronically injured TECs experience cell-cycle arrest which promote a paracrine EMT program in TECs, jointly leading to fibrosis. This platelet-mediated effect on cell cycle arrest and EMT was driven by TGF-β1 signaling, as selective inhibition of the TGF-β receptor rescued these dysfunctional phenotypes.

**Conclusion:**

Our study demonstrates that platelets activate the TGF-β1 pathway, leading to cell cycle arrest, EMT and renal fibrosis. These findings suggest that antiplatelet therapies may have potential renoprotective effects by protecting tubular homeostasis, attenuating partial EMT and fibrosis.

**Supplementary Information:**

The online version contains supplementary material available at 10.1186/s10020-025-01355-7.

## Introduction

Chronic kidney disease (CKD) is characterized by deterioration of kidney function as a result of progressive loss of functional kidney parenchyma and tissue fibrosis. The prevalence of CKD is increasing worldwide, partly due to the current global increased incidence of diabetes, with projections indicating CKD may become the fifth leading cause of death by 2040 (Francis et al. 2024). In addition to the rising prevalence, constrained therapeutic options becoming a major issue in the management of CKD patients (Wen et al. 2022).

Despite recent advances with therapies such as sodium-glucose co-transporter 2 inhibitors (SGLT2i) (Tsukamoto et al. 2022; Yau et al. 2022), mineralocorticoid receptor antagonists (Tsukamoto et al. 2022; Amornritvanich et al. 2024), and GLP-1 receptor agonists (Badve et al. 2025; Abasheva et al. 2024), which have demonstrated efficacy in slowing chronic kidney disease (CKD) progression, reducing kidney inflammation, and lowering the risk of cardiovascular events, there remains a critical unmet need for treatments that can fully restore kidney function or reverse established tissue fibrosis. While the role of EMT in renal fibrosis remains controversial, with lineage tracing studies disputing its contribution to the fibroblast pool, accumulating evidence suggests that epithelial plasticity and partial EMT may significantly contribute to the profibrotic environment during kidney injury (LeBleu et al. 2013; Loeffler and Wolf 2015; Gregorio et al. 2020). The main pathological feature of renal EMT is the gradual transformation of renal tubular cells from an epithelial into a mesenchymal phenotype, characterized by decreased E-cadherin, and increased vimentin and α-SMA expression. This transition results in the activation of myofibroblasts that produce extracellular matrix (ECM) proteins, leading to renal fibrosis and impaired renal tubular function (Nieto et al. 2016; Kalluri and Weinberg 2009). The term partial EMT was introduced based on recent evidence suggesting that renal TECs may not fully transition into fibroblasts by crossing the basement membrane. Following injury, these cells exhibit mesenchymal characteristics and can release various profibrotic proteins and cytokines while still remaining attached to the basement membrane (Lovisa et al. 2016; Grande et al. 2016). EMT program in TECs can be also driven by neighboring TECs subjected to repeated insults and chronic damage, resulting in cell cycle arrest. TECs arrested in the G2/M phase amplify the EMT and fibrotic response in the kidney through paracrine signaling. This creates a self-perpetuating cycle of EMT and cell cycle arrest in TECs, ultimately culminating in fibrosis (Lovisa et al. 2016).

There is growing evidence that platelet activation contributes to CKD progression by enhancing renal inflammation, oxidative stress, and fibrosis. Platelet activation in kidney disease may result from uremic toxin accumulation, which creates a state of chronic low-grade inflammation, as well as from endothelial dysfunction and vascular injury, both of which enhance platelet–endothelium interactions and activate coagulation pathways (Baaten et al. 2022; Gong et al. 2022). Platelet numbers and platelet activity correlate with a higher risk of cardiovascular disease in CKD stage 5 or dialysis patients (Gong et al. 2022). Previous studies from our group revealed a crucial role for platelets in promoting renal injury and fibrosis in models of acute kidney injury and DKD (Jansen et al. 2017; Uil et al. 2020). Platelet inhibition protected against the development of DKD in mice, accompanied by a reduction in kidney fibrosis. However, the role of platelets in (partial) EMT and CKD progression remains unknown so far. Understanding the molecular mechanisms of EMT and renal fibrosis in CKD remains a critical research focus to enable targeted therapies to decrease CKD progression. This study aims to fill the gap of knowledge and to explore the contribution of platelets in renal EMT mechanisms in different CKD models.

## Materials and methods

### Animal model

#### Mouse model of diabetic kidney disease

Male C57BL6/J mice, aged six weeks, were acquired from Charles Rivers Laboratories (France). Thirty-seven mice were randomized into three treatment groups: a control group (*n* = 9), a diabetic group without treatment (*n* = 14), and a diabetic group treated with a platelet inhibitor (*n* = 14), 3–4 animals per cage. Mice were housed in individually ventilated cages (IVCs) under a 12-hour light/dark cycle, with access to water and standard chow provided ad libitum for a duration of one week. Following their acclimatization, they underwent unilateral nephrectomy and either received a sodium citrate solution injection for non-diabetic controls or a 5-day intraperitoneal injection of streptozotocin (STZ, 50 mg/kg) to induce diabetes. One week after the final STZ dose, the mice were either given platelet inhibitor treatment or left untreated for a period of 16 weeks. Mice will receive either 100 µl saline or the platelet inhibitor ticagrelor (300 mg/kg) every two days. The mice in the control group, which did not have diabetes, were given saline as a vehicle treatment. The diabetic group comprising diabetic mice, received saline as a treatment. In the meantime, the treatment group consisting of diabetic mice were given Ticagrelor (300 mg/kg) as platelet inhibitor therapy. Bodyweight was monitored weekly and blood glucose levels (Contour next, Ascensia Diabetes Care, USA) were measured from tail vein weekly, until glucose levels stabilized. Mice that exhibited severe hyperglycemia (blood glucose levels > 30 mM) and experienced a weight loss of more than 15% in a week were administered 1/2 pellet of LinBit insulin (LinShin, Canada). Mice that did not reach blood glucose levels above 16 mM within two weeks received additional injections of STZ until their blood glucose levels reached at least 16 mM. At the end of the study, the mice were sacrificed, the right kidney was collected and divided into 2 parts, one section was fixed in 10% formalin, the rest was snap-frozen in liquid nitrogen.

#### Mouse model of unilateral ureter obstruction

Male C57BL6/J mice aged eight weeks (Charles River Laboratories, France) were randomized into two treatment groups: the platelet depletion group (*n* = 8) or the isotype control group (*n* = 6). Mice were housed in IVC cages with 12/12 light/dark cycle and received water and standard chow ad libitum. Following a week of acclimation, all of the mice then received complete ureteral blockage in the right kidney. Isoflurane (3–4% induction, 1.5–2.5% maintenance, 100% oxygen) was used as anesthetic drug. The ureter was accessed by making an abdominal incision, and it was twice ligated. 0.1 mg/kg subcutaneous injections of buprenorphine (Temgesic, Schering-Plough, the Netherlands) were given for analgesia before to surgery (0.5 h) and following surgery (6 h). Six hours following the operation, mice received daily intraperitoneal injections of non-immune control IgG (1 mg/kg/day, Emfret Analytic, Germany) or platelet-depleting anti-GP1b for 3 days. At the end of the study, mice were terminated under anesthesia and sacrificed by exsanguination via eye extraction, followed by cervical dislocation. The obstructed right kidney was collected and divided into 2 parts to be frozen, placed at −80 °C and the other part was fixed in 10% formalin.

### Cell culture

Renal tubular epithelial human cell line HK-2 were cultured in DMEM/F12 (Gibco) medium containing 10% Fetal Bovine Serum (FBS, Sigma Aldrich), 1% Penicillin-Streptomycin (Gibco), 1% glutamine (Gibco), 1% insulin-Transferin-Selenium (Gibco) and 1% mixed medium (Tri-iodo-thyronine (Sigma Aldrich), Hydrocortisone (Sigma Aldrich), epidermal growth factor (EGF, Sigma Aldrich)), at 37 °C under 5% CO_2_. After reaching confluency, cells were seeded at 50,000 cells/ml (6-well plates), 20,000 cells/ml (12-well plates) or 10,000 cells/ml (24 well plates with poly-L-lysine cover slip (Sigma-Aldrich)). Fresh platelets (resting or activated) were added every 3 days for 10 days. Three groups were studied: control (no treatment), activated platelets, and resting platelets. Experiments were performed in multiple independent biological replicates. The quantitative data presented represent biological replicates from different experiments.

### 3D Kidney organoid

Human induced pluripotent stem cells (iPSCs) were kindly provided by Dr. J. Jansen (Radboud University Medical Center, Nijmegen, the Netherlands) The Stem Cells Technology Center at Radboud University Medical Center applied the Yamanaka factors to reprogramme adult human skin fibroblasts from a healthy donor, generating these iPSCs.

The iPSC colonies were cultured in StemFlex medium (Thermo Fisher #A3349401) on 6-well plates pre-coated with Geltrex (Thermo Fisher #A1413301) and passaged with EDTA (Thermo Fisher #15575020). The differentiation process followed protocols established by Takasato et al. (Takasato et al. 2016) and Jansen et al. (Jansen et al. 2022). Differentiation was initiated on day 0 (d0) by treating the cells with 6 µM CHIR-99021 (R&D Systems #4423) in STEMdiff APEL2 medium (Stemcell Technologies #05270). In order to stimulate ureteric bud development, CHIR treatment was administered for 3 days, and 5 days for metanephric mesenchyme differentiation. After that, the medium was switched to APEL2 supplemented with heparin (1 µg/ml, Sigma-Aldrich #H4784) and fibroblast growth factor 9 (FGF9, 200 ng/ml, Peprotech #100 − 23) until day 7 (d7).

Cells were trypsinized with 0.05% trypsin-EDTA (Thermo Fisher, #25300) at day 7, and the 3-day and 5-day CHIR-differentiated cells were combined in a 1:2 ratio. Aggregates were formed by centrifuging 300,000 cells in 1.5-ml tubes at 300 rcf for 3 min in three consecutive cycles, rotating the tubes 180° between cycles. The aggregates were then transferred onto Costar Transwell filters (Corning #3450) and cultured at the air-medium interface to form 3D organoids. To promote nephrogenesis, a 1-hour pulse of 5 µM CHIR was applied in APEL2 medium, followed by an additional 5 days of culture in APEL2 medium supplemented with FGF9 and heparin. Starting on day 7 + 5, the organoids were cultured in E6 medium (Thermo Fisher #A1516401) containing human epidermal growth factor (10 ng/ml, Sigma-Aldrich #E9644), bone morphogenetic protein-7 (50 ng/ml, Peprotech #120-03P), and stromal derived factor 1 beta (10 ng/ml, R&D Systems #351-FS) until further experimental use.

### Platelet isolation

Venous blood from healthy volunteers (*N* = 4) was obtained using S-Monovette 10 ml 9NC tubes (Sarstedt) without using vacuum extraction. Platelet-rich plasma (PRP) was obtained by centrifugation of whole blood at 180 RCF for 15 min at 20 °C. Then PRP added with acid citrate dextrose (tri-Sodium citraat.2H_2_O 0.085 mol/L, glucose 0.11 mol/L, citric acid.H_2_O 0.071 mol/L) and centrifuge at 200 RCF for 20 min at 20 °C. Isolated platelets were resuspended in buffer A (NaCl 1_3_7 mmol/L, NaHCO_3_ 11.9 mmol/L, glucose 5.6 mmol/L, MgCl_2_.6H2O 1 mmol/L, KCL 2.61 mmol/L, EDTA 2 mmol/L) and centrifuged again in 200 RCF for 20 min at 20 °C. To prevent the activation, prostaglandin E1 (PGE1, 1 μm, Chem Cruz Biotechnology, Dallas, Tx) was added before each centrifugation. Platelet were counted using Guava EasyCyte HT Flow Cytometer (Merck Millipore) and resuspended in Buffer B (similar with Buffer A but without EDTA). Platelet activation was induced by stimulation with TRAP-6 (15 μm, Bachem, Germany) for 15 min at room temperature (RT).

### Immunohistochemical

Mouse kidneys were fixed in 10% formalin for 24 h and embedded in paraffin. Tissue sections were deparaffinized by xylene and rehydrated by ethanol. Endogen peroxidase was quenched with 3% hydrogen peroxidase (Sigma Aldrich) for 20 min at RT. Antigen retrieval was performed by boiling in sodium citrate buffer (pH 6.0). Sections were incubated overnight at 4 °C with primary antibodies: rabbit polyclonal IgG anti-SNAIL1 antibody (1:500, Santa Cruz, #sc.28199), mouse monoclonal IgG2aκ anti-αSMA (alpha smooth muscle actin) antibody (1:800, Dako, #M0851), mouse monoclonal IgG1 anti-Collagen 1 antibody (1:200, GeneTex, #GTX26308), rabbit monoclonal IgG anti-p21 antibody (1:1000, Abcam, #ab188224), and rabbit monoclonal anti-CD42b antibody (1:200, Abcam, #cloneSP219). After washing 3x with phosphate-buffered saline, the slides were incubated with biotinylated goat anti-rabbit secondary antibody (Bright Vision Immunologic, #DPVR110HRP) or goat anti-mouse secondary antibody (Bright Vision Immunologic, #DPVM110HRP) at RT for 30 min. The antibody staining was visualized with 3, 3’-diaminobenzidine (DAB) at RT for 15 min. Immunostained kidney sections were counterstained with Mayer’s hematoxylin to visualize cellular morphology. Identification of proximal tubular segment was performed by experienced nephropathologists, based on well-established morphological criteria such as epithelial cell shape, presence of a brush border, and tubular diameter. αSMA-, SNAIL1-, CD42b-, and p21-positive areas were quantified in 10 random fields at 20x magnification using ImageJ (v1.53, Win64, U.S. National Institutes of Health) or QuPath. SNAIL1 expression was scored in both the cortical and medullary regions in 10 random fields at 20x magnification using QuPath (version 0.4.3).

### Immunofluorescence

HK-2 cells were cultured on 12-mm glass coverslips in 24-well plates and treated as control, with resting platelets, or activated platelets every 3 days for 10 days. Cells were fixed with 4% paraformaldehyde (PFA) for 15 min, then permeabilized and blocked with 0.1% Triton X- 100 and 0.5% BSA in PBS for 30 min at RT. Samples were incubated overnight at 4 °C with the following primary antibodies: mouse monoclonal IgG2aκ anti-E-cadherin (1:1000, BD Biosciences, #610182), rabbit monoclonal IgG anti-SNAIL1 antibody (1:100, Cell Signaling, #3879), mouse monoclonal IgG2aκ anti-αSMA antibody (1:150, Dako, #M0851), and rabbit monoclonal IgG anti-p21 antibody (1:200, Abcam, #ab188224). After washing with blocking buffer, Alexa Fluor 488-conjugated donkey anti-rabbit IgG (1:500, Jackson, #711-548-152) or anti-mouse IgG (1:500, Thermo Fisher scientific, #A-21202) was applied for 1 h at room temperature. Following PBS washes, nuclei were stained with 1:2000 Hoechst 33342 (Hoechst 33342 Solution (20 mM); Invitrogen, Thermo Fisher Scientific).

For organoids, staining followed the method described by Jansen et al. (Jansen et al. 2022)). Organoids were washed twice with PBS, fixed in 2% PFA at 4 °C for 20 min, and rinsed three times with PBS. Blocking was performed with 10% donkey serum (GeneTex) and 0.6% Triton X-100 in DPBS for 2 h at room temperature. Organoids were incubated overnight at 4 °C with primary antibodies: rabbit polyclonal IgG anti-collagen I antibody (1:100, Abcam, #ab34710). After PBTX washes (0.3% Triton X- 100 in PBS), secondary antibodies Alexa fluor 594-conjugated goat anti rabbit IgG (1:400, Jackson ImmunoResearch, #711-586-152) were applied for 2 h at RT. Organoids were washed with DPBS and mounted with Aqua-Poly/mount (Polysciences, Warrington, PA, USA). Imaging was conducted using a Leica SPX8 confocal microscope.

### Reverse transcription quantitative PCR (RT-qPCR)

Total RNA was isolated from frozen kidney sections or cell culture using TRIzol Reagent (Invitrogen) according to the manufacturer’s instructions. For organoids, they were removed from the Transwell and placed in 300 µl of lysis buffer (PureLink RNA Mini Kit, Invitrogen, Cat. #12183018A) supplemented with 3 µl of β-mercaptoethanol (Sigma Aldrich), incubating for at least 1 h at −80 °C. RNA purification was performed according to the kit’s protocol.

cDNA was synthesized from 1 µg RNA using oligo-dT primers. The mRNA expression of relevant genes was analyzed by RT-qPCR using the LightCycler480 (Roche Diagnostics). For DKD and cell culture samples, gene expression was normalized against GAPDH, whereas for UUO and organoids, normalization was performed against TBP. The RT-qPCR mixture consisted of SYBR Green PCR master mix (Thermo Fisher), specific forward and reverse primers, and ddH2O. The RT-qPCR procedure followed the same steps for both tissue types. SYBR Green dye intensity was analyzed with linear regression analysis using LinRegPCR software. The primer pairs used are shown in Supplementary Table 1.

### Western blot analysis

Cells were lysed with RIPA buffer supplemented with phosphatase and protease inhibitors. Total protein concentration of the lysate was measured using the BCA Protein Assay Kit (Thermo Fisher, #23225). An equal amount (15 µg) of protein samples were electrophoresed on 4–12% Bis-Tris Plus Gels (Invitrogen, # NW04122BOX, #NW04127BOX) and transfer onto polyvinylidene difluoride membranes/PVDF transfer membrane (Thermo Fisher, #XL372851). Membranes were blocked with 5% skim milk/Tris-buffered saline with 0.1% (v/v) Tween^®^ 20 detergent (TBST) for 1 h at RT and incubated with primary antibody: mouse monoclonal IgG2aκ anti-E-cadherin antibody (1:1000, BD Biosciences, #610182), rabbit monoclonal IgG anti-SNAIL1 antibody (1:500, Cell Signaling, #3879), rabbit polyclonal IgG anti ZEB-1 antibody (1:500, Santa Cruz, #sc-25388), mouse monoclonal IgG2aκ anti-αSMA antibody (1:1000, Dako, #M0851), rabbit monoclonal IgG anti-p21 antibody (1:1000, Abcam, #ab188224), mouse monoclonal IgG anti-β-actin antibody (1:50000, Sigma Aldrich, #A1978) and mouse monoclonal IgG anti-β-tubulin antibody (1:1000, Sigma Aldrich, #T0198) at 4 °C overnight. After washing with TBST, membranes were incubated with HRP-conjugated secondary antibodies goat anti-rabbit IgG (1:2000, Dako, #P0448) or rabbit anti-mouse IgG (1:2000, Dako, #P0260) for 1 h. Signal detection was performed using an ECL Western Blotting Substrate Kit (Thermo Fisher, # 32106) and membranes were visualized with LAS 4000. We used ImageJ processing software (v1.53, win64, U.S. National Institutes of Health) to normalize and quantify the signals to those of proteins.

### Enzyme-linked immunosorbent assay (ELISA)

Culture medium from control and platelet-treated cells were collected every 3 days, coinciding with medium replace and fresh platelet addition. TGF-β level was measured using human TGF- β ELISA kit (R&D systems, #DY-240) according to the manufacturer’s instructions. Additional cytokine levels were measured using ELISA kits from R&D Systems: human IL-1β (#DY201-05), human TNF-α (#DY210-05), and human IL-10 (#DY217B-05). Platelet factor 4 (PF4) was measured using a combination of reagents from R&D Systems: monoclonal antibody (#MAB7951), biotinylated detection antibody (#BAF795), and recombinant standard (#795-P4). For all ELISA procedures, two-fold diluted samples were added to antibody-coated plates, followed by enzyme-labeled antibody incubating for 2 h. After five washes, the substrate for color development was added, and absorbance was measured at 450 nm using microplate reader (CLARIOstar).

### Flow cytometry

For flow cytometry, 5 µl of resting and activating platelet were added to a mixture of antibodies in HEPES buffer. These characterized by fluorescence activated cell sorting using platelet marker CD61-APC (1:10, Invitrogen, #MA5-23552) and platelet activating marker CD62P-FITC (1:20, Immunotech, #A07790). After 30 min incubation at RT in the dark, cells were fixed with 0.3% PFA in HEPES buffer for 1 h. Flow cytometry was performed using a BD™ Symphony flow cytometer. The number of resting platelets was determined by CD61 staining only, while activated platelet were marked by double staining of CD61 and CD62P. Data were analyzed using FlowJo (v10.5 Software; FlowJo LLC, Ashland, OR, USA). This analysis was performed solely to confirm whether the platelets were in a resting or activating state, and thus no quantitative data are presented.

### Statistical analysis

All data sets were tested for their distribution prior to analyses. Data with normal distribution were analyzed by unpaired Student T-test, while non-normally distributed data were analyzed with Mann-Whitney U test. For comparisons of more than two groups, one-way ANOVA or Kruskal-Wallis tests were employed, followed by post hoc tests for significant results. Data are presented as mean ± SEM (standard error of the mean), unless indicated otherwise in the figure legends. The threshold for significance was set at *p* < 0.05. All analyses were conducted using GraphPad Prism version 10.2.0 (GraphPad Software, San Diego, USA).

## Results

### Platelet inhibitor and depletion attenuates fibrosis by impeding partial EMT in kidney fibrosis models

**Fig. 1:**
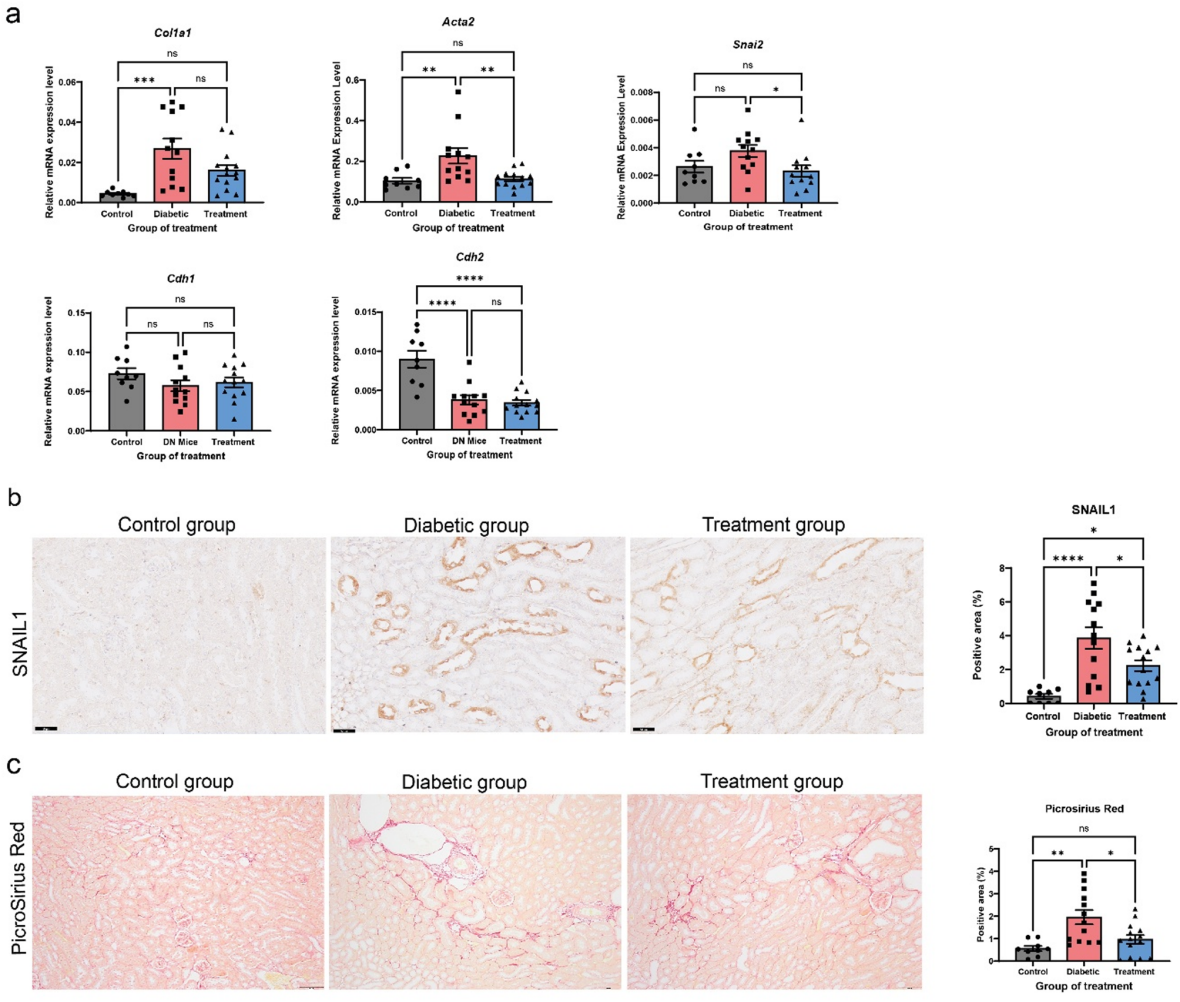
Ticagrelor treatment leads to a reduction in fibrosis, and this effect is associated with the inhibition of epithelial-mesenchymal transition (EMT) in mice with diabetic kidney disease (DKD). **A **Relative mRNA expression levels of *Col1a1*, *Acta2* (α-SMA), *Snai2* (Slug*)*, *Cdh1* (E-cadherin), and *Cdh2* (N-cadherin) in kidney tissues were assessed in the control group, DKD group, and DKD group treated with the platelet inhibitor Ticagrelor by RT-qPCR. **B** Representative images show SNAIL1 immunostaining in renal tissue sections from the same groups. The percentage of SNAIL1-positive cells was quantified from 10 non-overlapping fields per region. **C** Picrosirius Red (PSR) staining in renal tissue sections shows collagen deposition. Quantification shows the percentage of PSR-positive area across 10 non-overlapping fields. Scale bar, 50 μm. Data are presented as mean ± SEM. Statistical analysis was performed using One-way ANOVA for comparisons involving more than two groups, except for *Snai2* (graph A) which was analyzed using the Kruskal-Wallis test. *P < 0.05, **P < 0.01, ***P < 0.001 and ****P < 0.0001

In this study, animal models were used to investigate the role of platelets in partial EMT in kidney tissue using two renal fibrosis models, DKD and UUO. At the study endpoint, fasting glucose levels were measured to confirm the diabetic state in DKD mice. Diabetic mice exhibited markedly elevated glucose levels compared to controls (control: 7.5 ± 1.2 mM; diabetic: 23.1 ± 7.8 mM; diabetic + platelet inhibitor: 24.5 ± 3.1 mM), indicating sustained hyperglycemia consistent with the diabetic phenotype. As expected, kidney transcript expression of genes linked to the partial EMT and fibrosis were elevated in DKD mice, as evidenced by increased renal mRNA expression of *Col1a1* and *Acta2* (smooth muscle alpha-actin), along with decreased *Cdh2* (N-cadherin) and *Cdh1* (E-cadherin) levels. Treatment with platelet inhibitor Ticagrelor, resulted in decreased expression of *Acta2*,* Snai2*, but did not significantly restore *Cdh2* levels. *Cdh1* showed a slight, non-significant reduction in the diabetic group, which was also not reversed by platelet inhibition (Fig. 1A). At the protein level, immunohistochemistry showed elevated SNAIL1 and interstitial collagen deposition (as detected by Picrosirius Red [PSR]) in diabetic kidneys. Ticagrelor treatment reduced SNAIL1 expression and decreased PSR staining, indicating attenuation of tubular EMT and fibrosis (Fig. 1B-C). In contrast, α-SMA protein levels remained unchanged (Supplementary Figure S1). These findings are consistent with our previous report (Uil et al. 2020). Similarly, Collagen IV deposition showed a similar trend and is shown in Supplementary Figure S2.

**Fig. 2:**
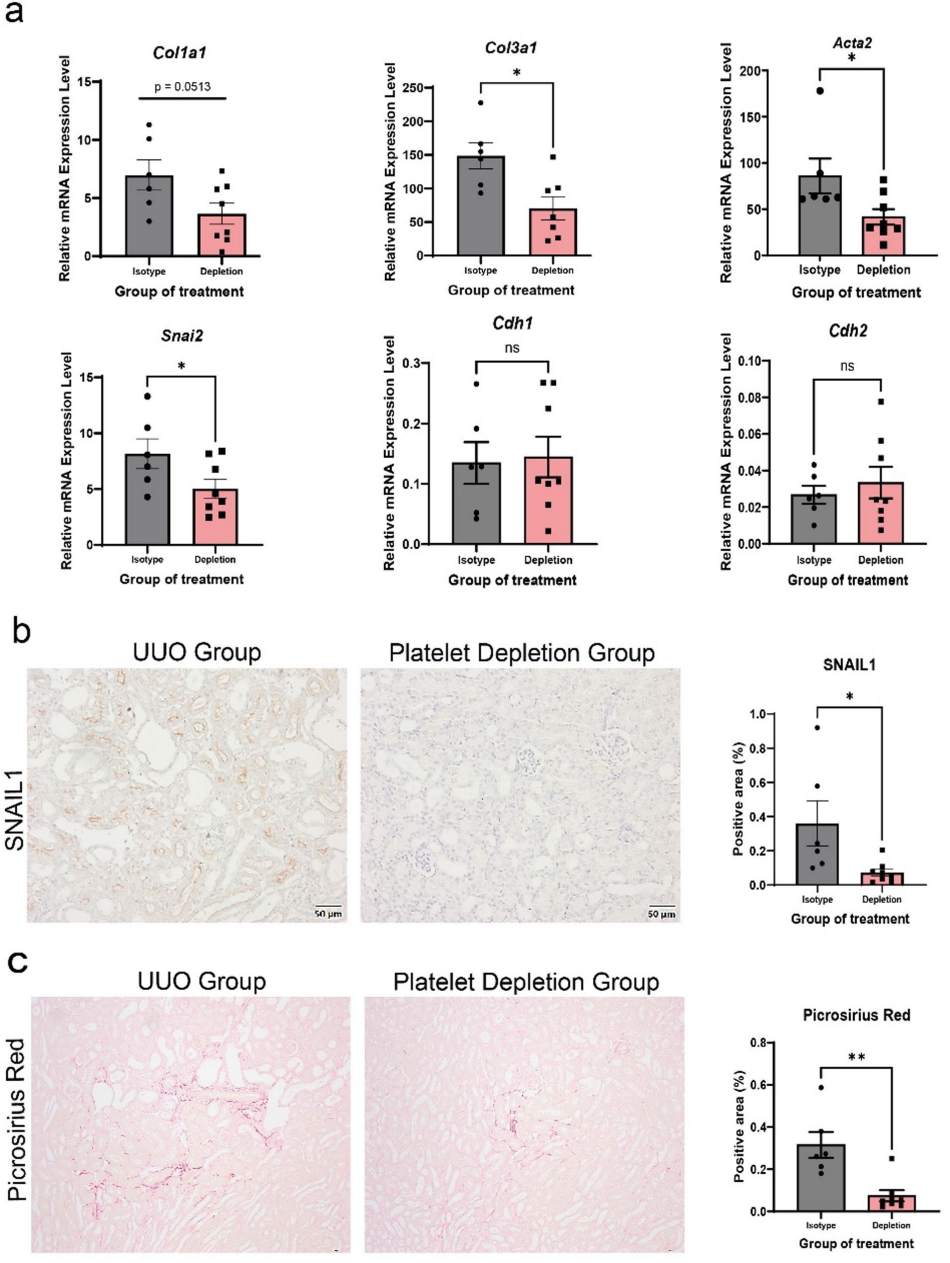
Platelet depletion attenuates fibrosis and abolishes partial EMT in UUO mouse model. **A** Relative mRNA expression levels of *Col1a1*,* Col3a1*,* Acta2*,* Snai2*, *Cdh1*, and *Cdh2* in the renal tissues from UUO isotype control compared with platelet depletion groups, measured by RT-qPCR. **B** Representative images show SNAIL1 staining in renal tissue section from UUO isotype control and UUO platelet depleted mice. The graph shows the percentage of SNAIL1-positive staining was quantified in 10 non-overlapping fields. **C** Picrosirius Red (PSR) staining shows collagen deposition, stained area were quantified similarly. Scale bar, 50 μm. Data are presented as mean ± SEM. Statistical analysis was performed using the Student’s *t*-test for comparison between two groups. *P < 0.05, **P < 0.01

In the UUO model, platelet-depletion with anti-GP1b antibodies led to a reduction in platelet count (Supplementary Figure S3). Consistent with the previous findings, we found a significant reduction in partial EMT markers including *Col1a1*, *Col3a1*, *Acta2* and *Snai2* in the platelet depletion group (Fig. 2A). Although *Cdh2* expression was not significantly restores, it showed a trend toward increased expression. Similarly, *Cdh1* also showed a non-significant upward trend in platelet depletion. Histological examination further showed a notable decreased in SNAIL1 positivity (Fig. 2B), collagen accumulation (PSR staining) (Fig. 2C) and α-SMA (Supplementary Figure S4) in the platelet depletion group.

Altogether, these finding suggest that platelets play a significant role in the development EMT and fibrosis. Platelet inhibition or depletion, can significantly ameliorate partial EMT in several models of renal fibrosis.

### Platelets induce partial EMT and fibrosis in proximal tubular epithelial kidney cells and kidney organoids

**Fig. 3:**
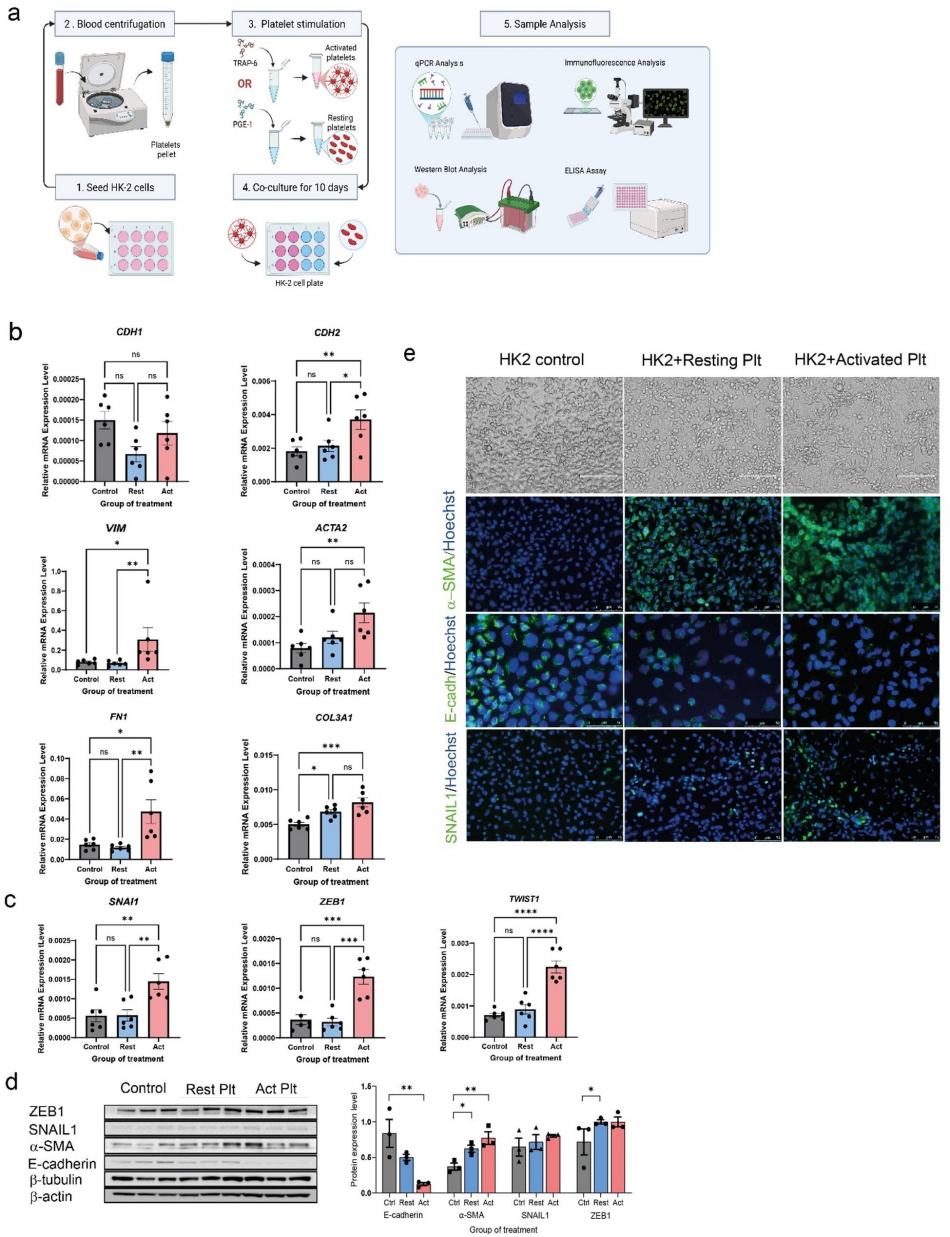
Platelets induce EMT in HK-2 cells. **A** This figure illustrates the in vitro experimental setup using renal HK-2 cells treated with either resting or activated platelets for 10 days. **B** The expression of epithelial and mesenchymal markers, as well as (**C**) EMT-related transcription factors, were quantified in HK-2 cell lysates using RT-qPCR. **D** Protein expressions were analyzed by immunoblotting and are presented as graphical results. **E** Morphological changes were analyzed by light microscopy in control HK-2 cells, resting platelet-treated cells, and activated platelet-treated cells. Representative immunofluorescence images show α-SMA, E-cadherin and SNAIL1 (green). Hoechst staining (blue) was used for nuclear visualization. Scale bars: 50 μm (α-SMA and SNAIL1) and 75 μm (E-cadherin). Data are presented as mean ± SEM. Statistical analysis was performed using One-way ANOVA for comparisons involving more than 2 groups, except for *Vim* (graph B) and ZEB1 (graph D), which were analyzed using the Kruskal-Wallis test. *P < 0.05, **P < 0.01, ***P < 0.001 and ****P < 0.0001

To explore the underlying mechanisms of platelet-induced partial EMT, we treated HK-2 proximal tubular epithelial cells with activated or resting platelets isolated from healthy volunteers. Platelet activation was induced using TRAP-6, while prostaglandin E1 was added to maintain platelets in a resting state and prevent activation. As illustrated in Fig. 3A, HK-2 proximal tubular epithelial cells were co-cultured with platelets over a 10-day period, with fresh platelets replenished every 3 days. The experimental groups included untreated controls, resting platelet-treated cells, and activated platelet-treated cells. Following the treatment period, we performed qPCR, immunostaining, Western blotting, and ELISA assay to assess changes in EMT-related markers and fibrosis. Compared to control, activated platelets-treated cells showed significant upregulation of mesenchymal markers, including *CDH2*, *FN*, *ACTA2*, *COL3A1*, and *VIM* (Fig. 3B), as well as EMT transcription factors (*SNAI1*,* ZEB1*, and *TWIST1)* (Fig. 3C). Conversely, the expression of epithelial marker *CDH1* (E-cadherin) was downregulated, although not statistically significant (Fig. 3B). Treatment with resting platelets resulted in minor changes in EMT markers without statistical significance. Western blot analysis confirmed the protein expression changes, showing a significant reduction in E-cadherin and an increase in α-SMA in activated platelet-treated cells, with trends of elevated SNAIL1 and ZEB1, although these were not statistically significant (Fig. 3D).

We also investigated the morphological changes induced by platelets in HK-2 cells. Initially, untreated cells exhibited a closely adherent, cuboidal morphology, characteristic of colonies with a classical cobblestone appearance. However, platelet-treated cells became separated from each other and adopted an elongated spindle-shaped, fibroblast-like morphology, further indicating EMT induction. Immunocytochemistry confirmed reduced E-cadherin and increased α-SMA and SNAIL1, with activated platelets having the strongest effect (Fig. 3E).

**Fig. 4:**
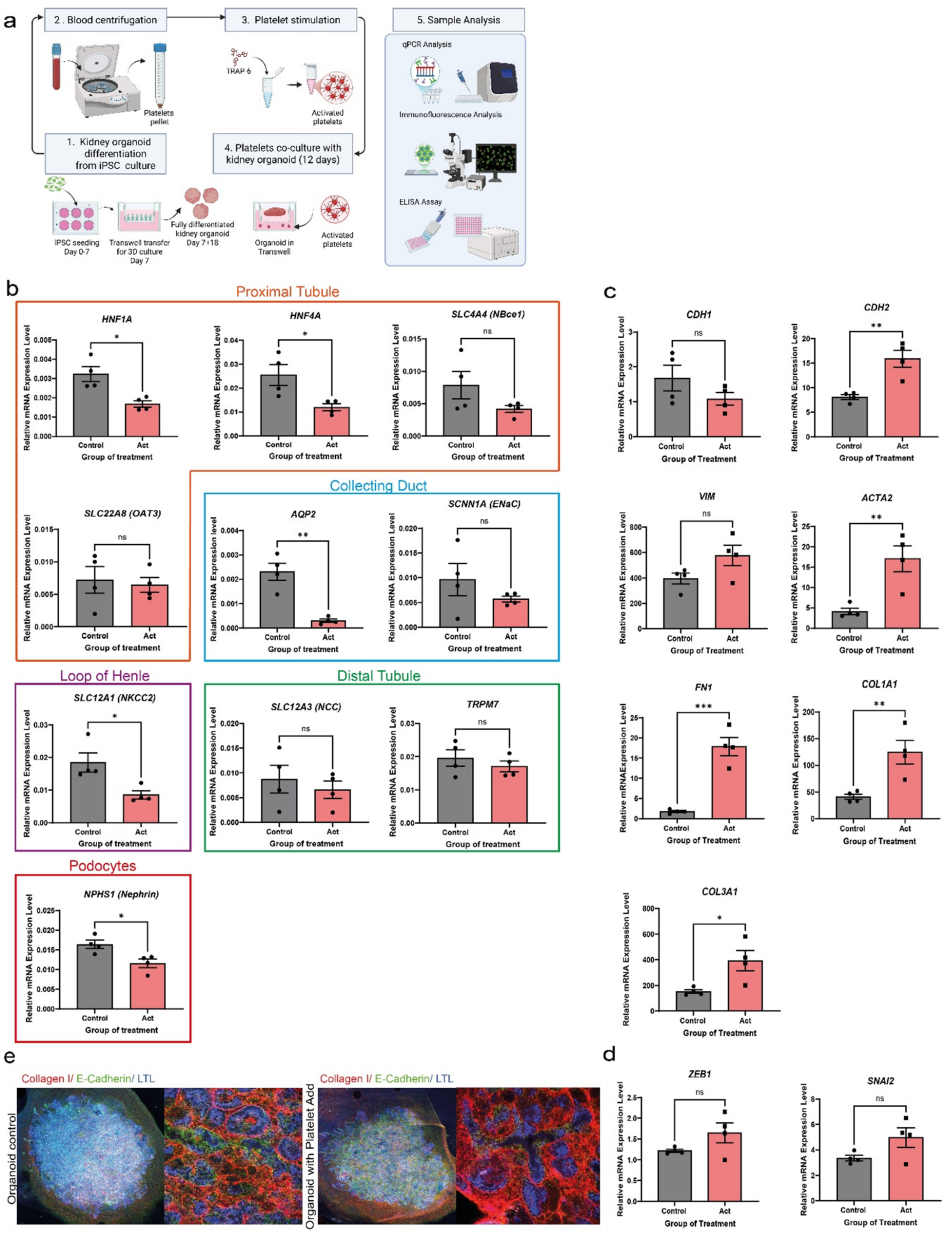
Platelets drive EMT and fibrosis in kidney organoids. **A** This figure illustrates the experimental setup in vitro using kidney organoids treated with activated platelets for 12 days. **B** The expression levels of tubule differentiation markers were measured by RT-qPCR. **C** The expression levels of epithelial, mesenchymal markers and fibrosis markers and (**D**) EMT-related transcription factors were quantified by RT-qPCR. **E** Representative immunofluorescence images shows LTL (blue), E-cadherin (green) and Collagen I staining (red) in control organoids and organoids with added platelets group. Scale bar, 25 μm (Collagen I). Data are presented as mean ± SEM. Statistical analysis was performed using the Student’s *t-*test for comparisons between two groups, except for *SLC12A1* (*NKCC2*) data, which were analyzed using the Mann-Whitney U test; *P < 0.05, **P < 0.01, and ***P < 0.001

We extended our analysis to kidney organoids derived from iPSCs generated using the protocol introduced by Jansen et al. (Jansen et al. 2022) These organoids contain renal structures, offering a relevant human 3D model for studying EMT. After full differentiation (Day 7 + 18), the organoids were stimulated with activated platelets using a Transwell co-culture system for 12 days, with platelets added to the basolateral compartment. Platelets and medium were refreshed every 3 days. Following the stimulation period, organoids were analyzed by qPCR, immunofluorescence staining, and ELISA to assess changes in gene expression, protein localization, and secreted factors associated with fibrosis and EMT (Figure.4 A). Given the significant effects observed with activated platelets in the 2D cell culture system, we focused exclusively on this group. Using the tubular cell differentiation markers identified by Yousef et al. (Yousef Yengej et al. 2023), we observed that stimulation with activated platelets in culture led to a reduction in differentiation markers of tubular cell types in iPSC-derived organoids compared to the control group, as demonstrated by bulk mRNA analysis (Figure.4B). Specifically, the expression of key expression markers was reduced including *HNF4α* for proximal tubular cells, *NKCC2* for thick ascending limb cells, *NCC* for distal tubule cells, *AQP2* for principal cells, and *Nephrin* for podocytes. Consistent with the observations in the 2D system, activated platelet-stimulated kidney organoids displayed significant alterations in EMT marker and fibrosis-related genes as demonstrated by increased expression of *ACTA2*,* FN1*,* CDH2*,* COL1A1* and *COL3A1* (Fig. 4C). *VIM*, *ZEB1* and *SNAI2* showed an increasing tendency following treatment with activated platelets, although this was not statistically significant (Fig. 4D). While light microscopy revealed no discernible morphological differences, immunofluorescence showed increased collagen I deposition, predominantly around tubular structures. Exposure to activated platelets induced early fibrotic changes in the organoid model, characterized by interstitial collagen I accumulation and partial loss of epithelial features. LTL staining remained detectable, indicating preserved proximal tubular identity, whereas E-cadherin expression was mildly reduced (Fig. 4E). Together with upregulation of mesenchymal markers (*ACTA2*,* FN1*,* CDH2*,* COL1A1* and *COL3A1*) and downregulation of *CDH1*, although this was not statistically significant, these findings suggest partial EMT-like changes in the tubular epithelium.

### Platelets activate TGF-β pathway and TGF-β inhibitor rescues platelet-induced partial EMT in HK-2 cells

**Fig. 5:**
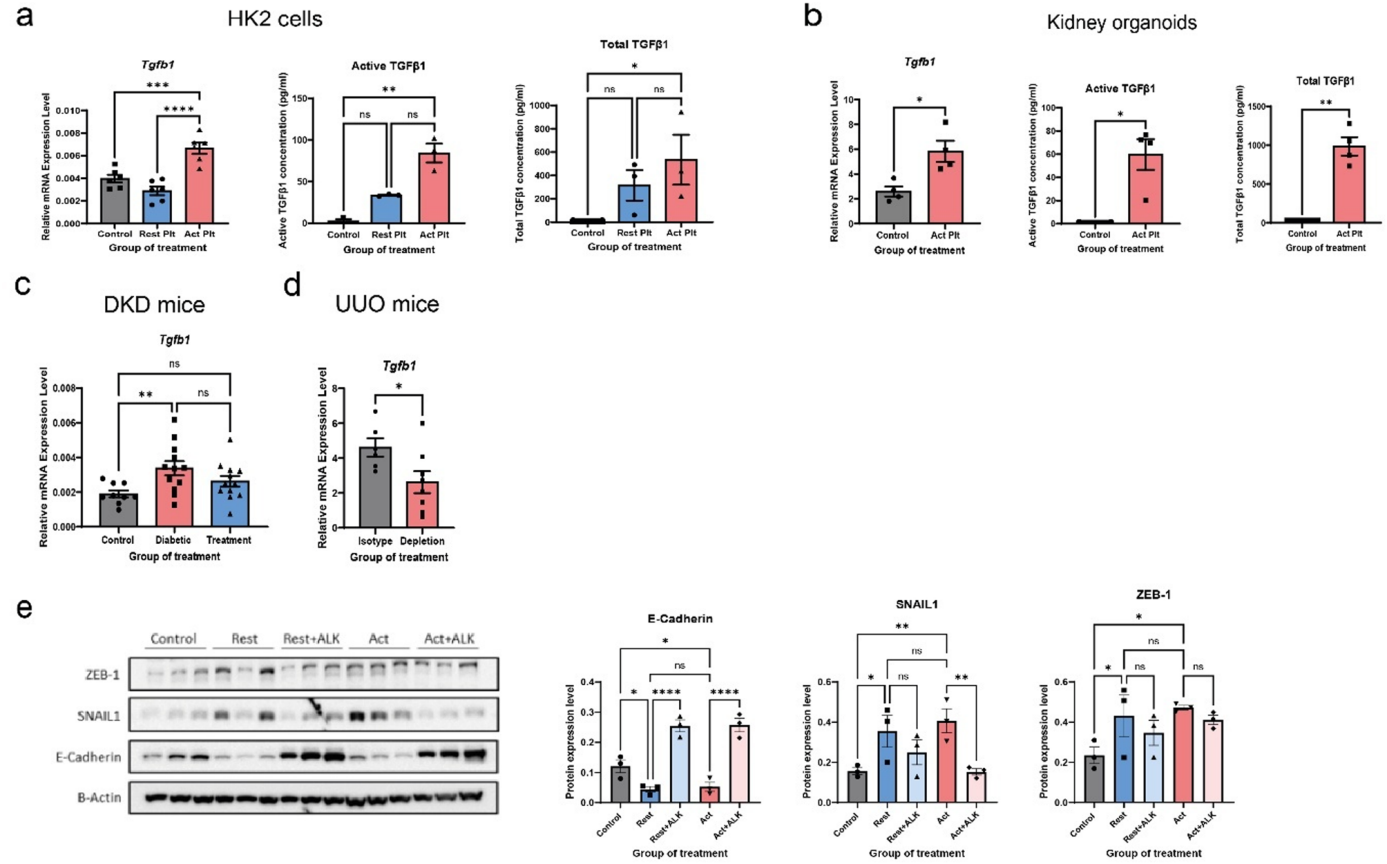
Platelets activate TGF-β pathway, and TGF-β inhibitor ameliorates platelet-induced EMT in HK-2 cells. **A**–**B** The concentration of active and total TGF-β in culture media was measured by ELISA, and relative mRNA expression levels were quantified by RT-qPCR in both HK-2 cells (after 10 days of platelet treatment) and kidney organoids (after 12 days of platelet treatment). **C**-**D** The relative mRNA expression level of TGF-β in cell lysates from DKD mouse model and UUO mouse model. **E** HK-2 cells were treated with resting or activated platelet for 10 days. The protein expressions levels of epithelial markers and EMT transcription factors were analyzed by immunoblotting, and reflected in graphs. Data are presented as mean ± SEM. Statistical analysis was performed using the Student’s *t*-test for comparisons between two groups, and One-way ANOVA for analyses involving more than 2 groups; *P < 0.05, **P < 0.01, ***P < 0.001, and ****P < 0.0001

Since platelets are known to induce TGF-β signaling, we hypothesized that platelet-induced EMT in HK-2 cells and kidney organoids could be mediated by TGF-β-pathway. To investigate this, we assessed both TGF-β1 gene expression and protein levels (total and active forms) following platelet stimulation. In HK-2 cells, TGF-β1 (*TGFB1*) mRNA was significantly upregulated after treatment with activated platelets compared to controls and resting platelets, indicating increased transcriptional activation of the cytokine. Similarly, ELISA measurements revealed that both total and active TGF-β1 protein levels increased in a stepwise manner: the control group showed very low or undetectable levels, resting platelets induced a moderate increase, and activated platelets led to the highest concentrations of both total and active forms of TGF-β1 (Fig. 5A).

A comparable pattern was observed in kidney organoids (Fig. 5B), where activated platelet stimulation led to a marked increase in *TGFB1* mRNA, total and active forms of TGF-β1 protein levels compared to controls. These findings suggest that platelet stimulation enhances TGF-β1 transcription, release, and extracellular activation—resulting in increased levels of the biologically active cytokine. On the other hand, in both DKD and UUO models, platelet inhibition or depletion also led to a reduction in *TGF-β1* mRNA levels (Fig. 5C-D).

To further validate the role of the TGF-β, platelet-treated HK-2 cells were cultured with or without the TGF-β type 1 receptor inhibitor ALK-5 kinase (10µM). There was downregulation of E-cadherin and upregulation of EMT markers (SNAIL1 and ZEB1) in platelet-treated HK-2 cells, while ALK-5 restored E-cadherin expression and suppressed SNAIL1 expression. ZEB1 expression was also reduced, although the change was not statistically significant (Fig. 5E), suggesting that platelet-induced EMT is dependent on the activation of the TGF-β1 pathway.

In addition to TGF-β1, we measured a panel of proinflammatory cytokines in the HK-2 cells supernatant, including IL-1β, IL-10, and TNF-α. All cytokines remained at low or undetectable levels in the supernatant of platelet-stimulated group compared to control. Meanwhile, PF4 levels were elevated in the supernatant following platelet stimulation, correlating with TGF-β1 upregulation (Supplementary Figure S5).

### Platelet-activated TGF-β pathway leads to the upregulation of p21 expression

**Fig. 6:**
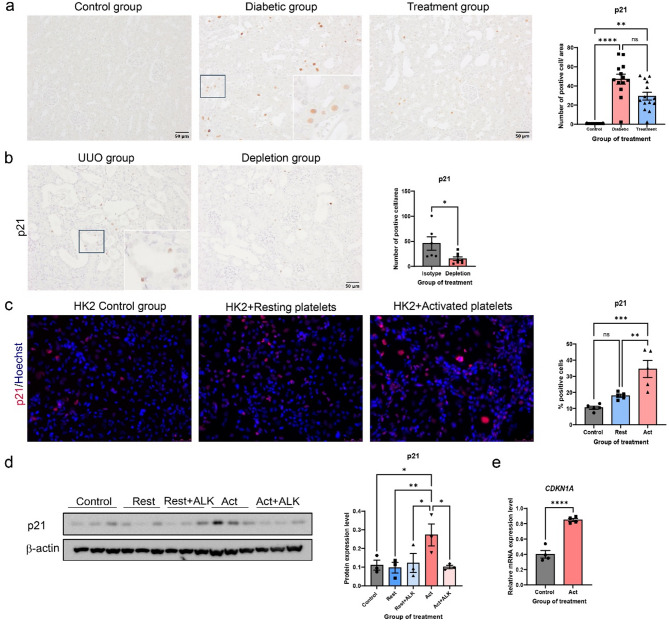
Platelets activate TGF-β pathway, accompanied by the upregulation of p21. **A**-**B** Representative images show p21 staining in renal tissue sections from the DKD and UUO mouse model. Insets highlight high-magnification views of tubular epithelial cells exhibiting positive p21 staining. The graph shows the percentage of p21-positive staining in the DKD mouse model in 10 non-overlapping fields. **C** Representative immunofluorescence images demonstrate p21 staining (red) in HK-2 cells. The graph shows the percentage of p21-positive staining from HK-2 cells in 10 non-overlapping fields. **D** Protein expressions levels of p21 were analyzed by immunoblotting. The graph presents the results from immunoblotting. **E** Transcript expression of *CDKN1A* in kidney organoids. Scale bar, 50 μm. Data are presented as mean ± SEM. Statistical analysis was performed using the Student’s *t*-test for comparisons between two groups, and One-way ANOVA for analyses involving more than 2 groups, except for p21 data from the DKD mice model (graph A), which were analyzed using the Kruskal-Wallis test; *P < 0.05, **P < 0.01, ***P < 0.001, and ****P < 0.0001

In CKD, partial EMT and cell cycle arrest are pivotal in driving renal injury and fibrosis progression. We explored the role of platelets in promoting cell cycle arrest via the upregulation of p21, a key regulator of cell cycle arrest at the G2/M checkpoint. In DKD mice, the diabetic group displayed notably elevated renal p21 + cells compared to the control group (Fig. 6A). Administration of the platelet inhibitor led to a trend in decrease p21 + cells expression. Similarly, in the UUO model, platelet depletion reduced p21 + cells compared to control (Fig. 6B).

Furthermore, HK-2 cells and kidney organoids treated with activated platelets exhibited increased p21 expression (Fig. 6C and E). TGF-β inhibitor ALK-5 downregulated p21 protein levels in HK-2 cells treated with activated platelets (Fig. 6D). These finding suggest that activated platelets not only promotes EMT in TECs but also contribute to the progression of renal fibrosis by inducing p21-mediated cell cycle arrest in response to TGF-β, a potent inducer of EMT and fibrosis.

## Discussion

Emerging evidence highlights the pivotal role of platelets in CKD progression. In this study, we demonstrated that platelet activation downregulated CDH1, upregulated mesenchymal markers (FN, VIM, ACTA2, CDH2, COL1A1 and COL3A1) and EMT transcriptional factors (SNAI1, SNAI2, ZEB1, and TWIST1). This aligns with the concept of partial EMT, where cells display the characteristics of mesenchymal cells and release a number of profibrotic proteins and cytokines, but remain attached to basement membrane (Lovisa et al. 2016).

By employing two distinct injury models (DKD and UUO) and anti-platelet strategies, we demonstrated that platelet involvement in renal EMT and fibrosis is relevant in both chronic and acute settings. Despite differences in timing and underlying mechanisms, these models provide complementary insights into the pathogenic role of platelets. Importantly, in our previous work using an ischemia-reperfusion injury (IRI) model, we showed that platelet activation contributes to acute tubular damage, and that its inhibition attenuates inflammation and necrosis (Jansen et al. 2017), further supporting a causal role for platelets across various forms of kidney injury.

As part of this study, we used the isotype control group (obstructed kidney with vehicle treatment) as the baseline comparator in the UUO model to assess the specific effects of platelet depletion. This design, enabled us to evaluate treatment-related changes within a disease context. While a normal, non-obstructed kidney control was not included, we acknowledge that its presence could have provided additional physiological context to better appreciate the magnitude of UUO-induced changes.

In animal models, we found that platelet activation promotes mesenchymal and fibrotic responses in kidney injury, evidenced by increased expression of *Snai2*,* Acta2*,* Col1a1*, and *Col3a1*, along with accumulation of collagen, α-SMA and SNAIL1 proteins. Platelet depletion or inhibition with ticagrelor attenuated these responses, reducing mesenchymal gene expression and collagen deposition. However, in the DKD model (Supplementary Figure S1), α-SMA protein levels did not significantly decrease despite marked reductions in *Acta2* and *Col1a1* transcripts. This dissociation likely reflects the chronic nature of DKD, where slow cellular turnover and persistent myofibroblast activation allow α-SMA protein to persist even after upstream signaling is suppressed (Kramann et al. 2013; Duffield 2014). Supporting this, PSR staining showed reduced collagen accumulation, indicating that while α-SMA⁺ cells remain, their fibrogenic activity may be diminished by platelet inhibition.

In assessing epithelial changes, we evaluated *Cdh2* and *Cdh1* expression. In the mouse kidney, Cdh2 (N-cadherin) is primarily expressed in proximal tubules, whereas Cdh1 (E-cadherin) is confined to distal segments and minimally expressed proximally (Prozialeck et al. 2004; Keller et al. 2012; Terada et al. 2017). Both markers showed a downward trend in diabetic kidneys, consistent with epithelial injury. Treatment with ticagrelor or platelet depletion led to only modest, non-significant increases in expression, suggesting limited reversibility of epithelial features in the chronic DKD setting. Together, these findings highlight the central role of platelet activation in driving mesenchymal and fibrotic remodeling, and demonstrate that platelet inhibition can partially attenuate these processes—though reversal of established epithelial changes may be constrained in chronic disease.

In our in vitro experiments, we used HK-2 cells derived from the proximal tubule, which express CDH1 under culture conditions—a pattern not fully reflective of in vivo expression in human kidneys (Slusser et al. 2015; Choi et al. 2016). The observed changes in CDH1 in our experiments, therefore, should be interpreted within the context of this in vitro adaptation. Nevertheless, the downregulation of CDH1, along with changes in other EMT markers, supports a phenotypic shift consistent with EMT in this cell system. Importantly, the observed EMT-like changes, including the downregulation of CDH1 and upregulation of CDH2 and other mesenchymal markers, were induced by activated platelet stimulation, highlighting the functional role of platelets in promoting EMT in this model. This shift from E-cadherin to N-cadherin expression, often referred to as cadherin switching, is a well-established hallmark of EMT and is typically accompanied by spindle-shaped morphological changes, further supporting the occurrence of EMT in our system (Loh et al. 2019).

Consistent with the in vivo findings, our in vitro experiments demonstrated that stimulation with resting platelets also induced upregulation of mesenchymal marker and EMT-related transcription factors, but to a lesser extent than activated platelets. Although resting platelets are considered quiescent, they still contain numerous bio-active molecules, stored in granules. Prolonged co-culture conditions may subtly alter this inactive state, leading to mesenchymal changes in epithelial cells.

During the process of EMT, the loss of functional E-cadherin-mediated cell-cell adhesion is consistently observed and serves as a critical biochemical marker of EMT process. The mechanisms underlying the alteration of EMT is driven by transcription factors such as *SNAI1*,* SNAI2*,* TWIST1*, and *ZEB1*, which regulate genes involved in the transition to a mesenchymal phenotype (Thiery et al. 2009; Yang and Weinberg 2008; Peinado et al. 2003). The studies by Lovisa et al. (Lovisa et al. 2015) and Grande et al. (Grande et al. 2016), demonstrated that deletion of *Twist1* or *Snai1* in mouse models of renal fibrosis inhibits the EMT and reduces fibrosis. *Zeb1* also plays a role in EMT in liver fibrosis and its inhibition reduce the EMT (Zhao et al. 2020). As direct repressors of E-cadherin, SNAIL1, ZEB1, they bind to the E-box located in the *CDH1* promoter. While *TWIST1* is primarily recognized for its ability to suppress *CDH1* indirectly, it can also directly bind to E-boxes 2 and 3 of the *CDH1* promoter to limit its expression (Serrano-Gomez et al. 2016). These genes involved in promoting EMT, a process that is central to various physiological and pathological events, including development, wound healing, and cancer metastasis (Thiery et al. 2009).

Furthermore, TGF-β family members are often implicated in EMT processes. Notably, in the context of renal fibrosis, TGF-β1 is a critical regulator of EMT (Peinado et al. 2003; Zeisberg and Kalluri 2004). We observed elevated TGF-β1 levels in the culture medium of HK-2 cells and kidney organoids treated with activated platelets, suggesting that platelet-released TGF-β1 affects surrounding cells. Using a TGF-βRI inhibitor (ALK-5 kinase), we found reversal of partial EMT, marked by restored E-cadherin and suppressed α-SMA, SNAIL1 and ZEB1 expression in platelet-treated HK-2 cells. This implies that platelet-induced partial EMT is mediated via the TGF-β signaling, a key regulator of EMT and fibrosis.

In this study, we also found that in a more complex human model such as kidney organoids, stimulation with activated platelets significantly reduced tubular differentiation markers. Platelet activation may have a major impact on tubule differentiation indicators in organoids, primarily due to the release of a variety of bioactive molecules, including TGF-β that change cellular activity and organoid development. This growth factor disrupts tubule differentiation by driving extracellular matrix deposition, EMT, and myofibroblast activation, causing tissue remodeling (Khan et al. 2023; Yun et al. 2016; Deng et al. 2024; Zhang et al. 2021).

Platelets contain significant reservoirs of TGF-β within their α-granules and are the primary source of TGF-β levels in the bloodstream. Research indicates a direct association between platelet count and peripheral TGF-β levels. Platelets not only release TGF-β but also respond to its signals via TGF-β type II receptors. Furthermore, TGF-β activates the protein Smad2. This positions platelets as key modulators of TGF-β, influencing various physiological and pathological processes (Lev et al. 2007; Karolczak and Cezary 2021).

Research related to platelets and EMT has been carried out in the field of malignancies, where platelets drive EMT through the TGF-β signaling, as seen in ovarian cell cancer (Guo et al. 2019) and bladder squamous cell carcinoma (Takemoto et al. 2017). In the context of renal fibrosis, blocking TGF-β signaling is a promising therapeutic approach to mitigate fibrotic changes and preserve renal function. TGF-β inhibitors have been extensively studied in both preclinical and clinical settings for their potential to alleviate renal fibrosis, improving renal function, and decreasing proteinuria in various CKD models, including UUO (Moon et al. 2006) and DKD (Benigni et al. 2006). Although there are disparities between successful preclinical results and clinical applications, the potential therapeutic benefits of antifibrotic treatments, capable of reducing or even reversing CKD progression in the future, is still very promising (Park and Yoo 2022).

In addition to TGF-β1, we assessed a panel of proinflammatory cytokines in the supernatant following platelet stimulation, including IL-1β, IL-10, and TNF-α. Among these, most showed low or undetectable levels in the supernatant of platelet-stimulated group compared to control (Supplementary Figure S5). This finding suggest that TGF-β1 emerged as the key mediator driving the epithelial-to-mesenchymal transition in response to activated platelets.

Although direct evidence linking PF4—a chemokine released from platelet alpha granules and a marker of platelet activation—to EMT in the kidney remains limited, emerging studies have begun to uncover its broader role beyond coagulation and inflammation. Recent findings suggest that PF4 may contribute to fibrotic remodeling and EMT-related processes, partly through enhancement of TGF-β signaling (Xiao et al. 2024; Cao et al. 2024). In our study, elevated PF4 levels in the supernatant of platelet-stimulated cultures were accompanied by increased TGF-β1 expression at both the transcript and protein level, along with upregulation of mesenchymal and EMT-related transcriptional markers, further strengthening the link between platelet-derived PF4, TGF-β activation, and EMT-like changes in kidney injury.

It is important to note that under physiological conditions, platelets do not directly interact with renal tubular epithelial cells due to the endothelial barrier. Instead, platelet influence on tubular epithelium is likely mediated indirectly through soluble factors such as cytokines, growth factors, and platelet-derived microvesicles released during inflammation or injury (Gong et al. 2022; Rustiasari and Roelofs 2022). Our in vitro model using Transwell co-culture further reflects this indirect mode of interaction, as platelets and kidney organoids are physically separated, allowing only soluble mediators to affect tubular cells. This highlights the relevance of paracrine signaling pathways in platelet-driven renal injury and fibrosis.

Recent findings suggest a relationship between EMT and cell cycle regulation in CKD. Cell cycle arrest, characterized by the overexpression of cell cycle inhibitors like p21, has been connected to EMT induction in both human kidneys and mouse model of renal fibrosis (Qi et al. 2021). Our study highlights that platelet-induced upregulation of p21 drives cell cycle arrest, which exacerbates fibrosis. Platelet inhibitors reduced p21 expression in both mouse models and in vitro, indicating that targeting the platelet-TGF-β-p21 axis may offer a new therapeutic strategy. Cell cycle inhibitors, such as p21 are elevated due to altered gene expression during EMT induction, leading to cell cycle arrest. TGF-β, a key regulator of EMT, causes p21 production, which inhibits cyclin-dependent kinases and results in cell cycle arrest (Jansen et al. 2017). On the other hand, cell cycle arrest might enhance susceptibility to EMT signals mediated by TGF-β. Prolonged cell cycle arrest can change the cellular milieu, triggering molecules that promote EMT. Antiplatelet therapies, commonly used for cardiovascular protection in CKD, may thus also have primary renoprotective effects, preserving tubular homeostasis attenuating EMT and fibrosis.

While our study provides evidence of partial EMT through the identification of key transcriptional regulators such as SNAIL by immunohistochemistry, integrated at the tissue level with morphological and spatial context, we acknowledge the inherent limitations of this approach. Future studies employing spatially resolved single-cell techniques would be highly valuable for more precisely mapping the cellular heterogeneity and dynamic transitions associated with partial EMT in this context.

## Conclusion

Our study highlights the role of platelets in CKD progression, demonstrating their contribution to renal fibrosis through TGF-β1–mediated EMT and cell cycle arrest. These findings suggest that targeting platelet-driven TGF-β1 signaling may offer a promising therapeutic approach to attenuate fibrosis and preserve kidney function. While antiplatelet agents are commonly used in CKD to reduce cardiovascular risk, their impact on kidney fibrosis remains underexplored. Our results indicate that platelet inhibition could provide dual benefits—cardiovascular protection and fibrosis attenuation. Further research is needed to translate these preclinical insights into clinical applications and optimize antiplatelet strategies for CKD management.

## Supplementary Information

Supplementary Material 1.

## Data Availability

No datasets were generated or analysed during the current study.

## Abbreviations

CKD: Chronic kidney disease
EMT: Epithelial-to-mesenchymal transition
DKD: Diabetic kidney disease
UUO: Unilateral ureter obstruction
TGF-β: Transforming growth factor beta
TECs: Tubular epithelial cells
ECM: Extracellular matrix
IVC: Individually ventilated cages
STZ: Streptozotocin
RT: Room temperature
αSMA: Alpha smooth muscle actin
PSR: Picrosirius Red
PBS: Phosphate-buffered saline
DAB: 3, 3’-diaminobenzidine
PFA: Paraformaldehyde
PRP: Platelet-rich plasma
iPSCs: Induced pluripotent stem cells
FGF9: Fibroblast growth factor 9
RT-qPCR: Reverse transcription quantitative PCR
ELISA: Enzyme-linked immunosorbent assay
