# Supplementary material for: Platelets induce epithelial to mesenchymal transition in renal proximal tubular epithelial cells through TGF-β signaling pathway
Source: Mol Med. 2025 Oct 29;31:318. doi: 10.1186/s10020-025-01355-7 (PMC12570672; doi:10.1186/s10020-025-01355-7)
Supplement: Supplementary file 1 — Supplementary Material 1. [file 10020_2025_1355_MOESM1_ESM.docx]

**Supplementary Table S1.**

**Table S1. qPCR primer sequences**

| Gene | Forward | Reverse |
| --- | --- | --- |
| Mouse primers |  |  |
| SLUG | TGTATGATGCCTGGTTGT | GTTTGTCTTTCCCTCCTC |
| α-SMA | CTGACAGAGGCACCACTGAA | AGAGGCATAGAGGGACAGCA |
| Coll1A1 | GCTCTTTTTAGATACTGTGGTGAGGAA | GTTTCCACGTCTCACCATTG |
| Coll3A1 | ACAGCTGGTGAACCTGGAAG | ACCAGGAGATCCATCTCGAC |
| TGF-β | ATACGCCTGAGTGGCTGTCT | TAATACGACTCACTATAGGGGCTTGCCACCCACGTAGTAG |
| GAPDH | TGTCCGTCGTGGATCTGAC | CCTGCTTCACCACCTTCTTG |
| TBP | GGAGAATCATGGACCAGAACA | GATGGGAATTCCAGGAGTCA |
| Human primers |  |  |
| SNAIL1 | GGACCCACACTGGCGAGAAG | TGTGGAGCAGGGACATTCGG |
| SLUG | CCCTGGTTGCTTCAAGGACA | AATGCTCTGTTGCAGTGAGGG |
| ZEB-1 | GGCATACACCTACTCAACTACGG | TGGGCGGTGTAGAATCAGAGTC |
| TWIST | AGCAAGATTCAGACCCTCAAGCT | CCTGGTAGAGGAAGTCGATGTACCT |
| E-Cadherin | GGGCTGGACCGAGAGAGTTT | GGGGGCATCAGCATCAGTCA |
| N-Cadherin | CCTCCAGAGTTTACTGCCATGAC | GTAGGATCTCCGCCACTGATTC |
| α-SMA | ACTGCCTTGGTGTGTGACAA | CACCATCACCCCCTGATGTC |
| Vimentin | GACGCCATCAACACCGAGTT | CTTTGTCGTTGGTTAGCTGGT |
| Collagen 1 | AGGTGAAGCAGGCAAACCT | CTCGCCAGGGAAACCTCT |
| Collagen 3A | GGTGGTTTTCAGTTTAGCTAC | CAGTGTGTTTCGTGCAAC |
| Fibronectin | GCGAGAGTGCCCCTACTACA | GTTGGTGAATCGCAGGTCA |
| P21 | AGGTGGACCTGGAGACTCTCAG | TCCTCTTGGAGAAGATCAGCCG |
| TGF-β | GCAGCACGTGGAGCTGTA | CAGCCGGTTGCTGAGGTA |
| GAPDH | CCGCATCTTCTTTTGCGTCG | AGTTAAAAGCAGCCCTGGTGA |
| TBP | CCCATGACTCCCATGACC | TTTACAACCAAGATTCACTGTGG |
| AQP2 | CTCCATGAGATCACGCCAGC | TCATCGGTGGAGGCGAAGAT |
| SCNN1A (ENaC) | GCAGACGCTCTTTGACCTGTA | AGTCCGATTTGTTCTGGTTGC |
| HNF1A | CCAGTAAGGTCCACGGTGTG | TTGGTGGAGGGGTGTAGACA |
| HNF4A | CACGGGCAAACACTACGGT | TTGACCTTCGAGTGCTGATCC |
| SLC4A4 (NBce1) | TCTCCAGTGCAAGTAGGATGT | GGTCCTTCTCCGGTTTATCAGA |
| SLC22A8 (OAT3) | CCAGAGTCCATACGCTGGTTG | TCACTTGCGGTGTACTTGGC |
| SLC12A1 (NKCC2) | AACTTTGGGCCACGCTTCAC | CCACACAGGCCCCTACACAA |
| SLC12A3 (NCC) | CTCCACCAATGGCAAGGTCAA | GGATGTCGTTAATGGGGTCCA |
| TRPM7 | TCCATTTACACCTGTGCCTCC | GCTCTTCGTAAACCTCCTCCC |
| NPHS1 (Nephrin) | GTCACCTTTCCCCCTAGTGC | AGCCACCATCGTAGCAGAAC |
| RPLPO | TCGACAATGGCAGCATCTAC | ATCCGTCTCCACAGACAAGG |


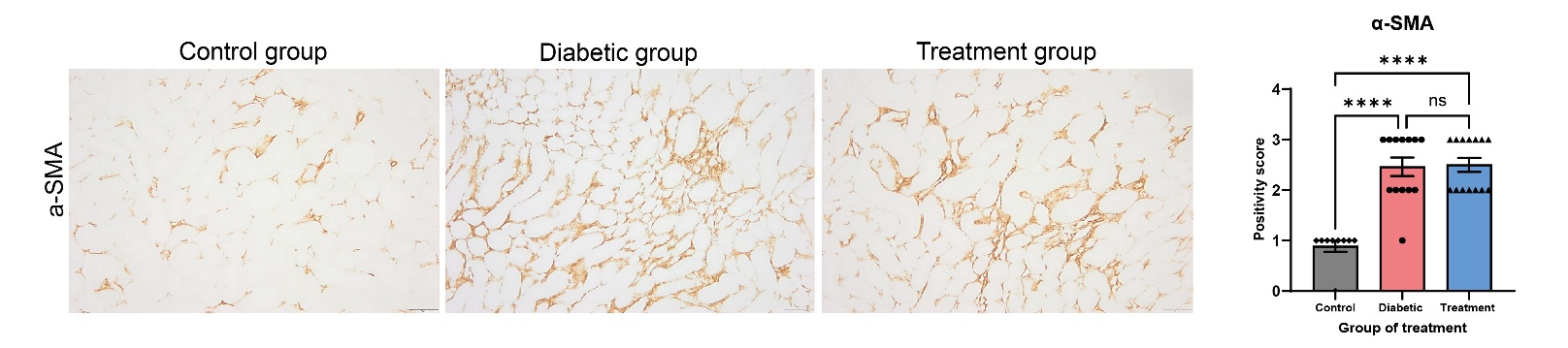
**Supplementary Figure S1.**

**Figure S1.** **α-SMA staining in DKD mouse kidney.** Representative images show α-SMA immunostaining in renal tissue sections from the control, DKD, and Ticagrelor-treated groups. Staining was semi-quantitatively scored based on the proportion of positively stained cells or area: 0 = negative, 1 = 1–25%, 2 = 26–50%, 3 = 51–75%, and 4 = >75%. Scale bar, 50 µm. Data are presented as mean ±SEM. Statistical analysis was performed using Kruskal-Wallis test for comparisons involving more than two groups. ****P<0.0001


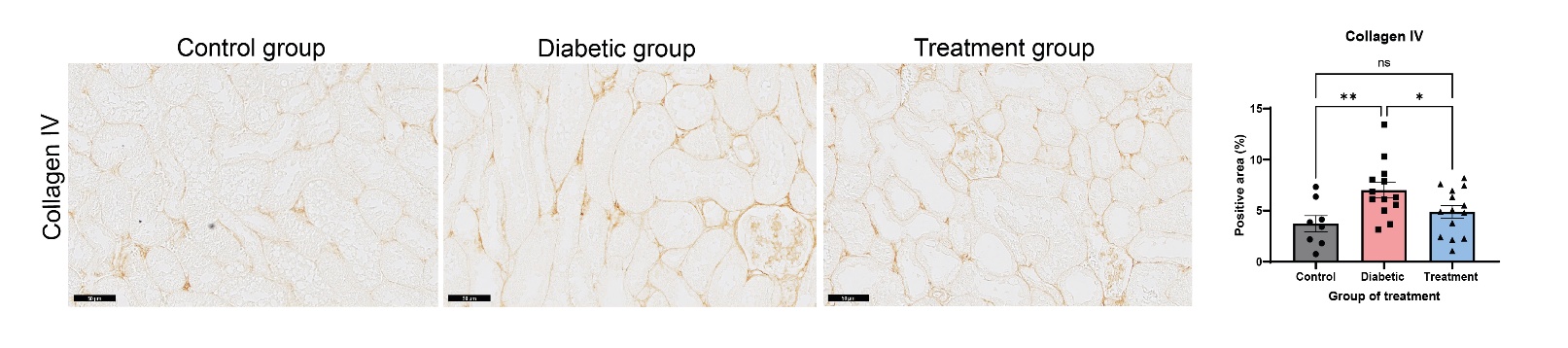
**Supplementary Figure S2.**

**Figure S2.** **Collagen IV staining in DKD mouse kidney.** Representative images show Collagen IV immunostaining in renal tissue sections from the control, DKD, and Ticagrelor-treated groups. The graph shows the percentage of Collagen IV-positive staining was quantified in 10 non-overlapping fields using FIJI image analysis software. Scale bar, 50 µm. Data are presented as mean ±SEM. Statistical analysis was performed using One-way ANOVA for comparisons involving more than two groups. *P<0.05, **P<0.01

**Supplementary Figure S3.**


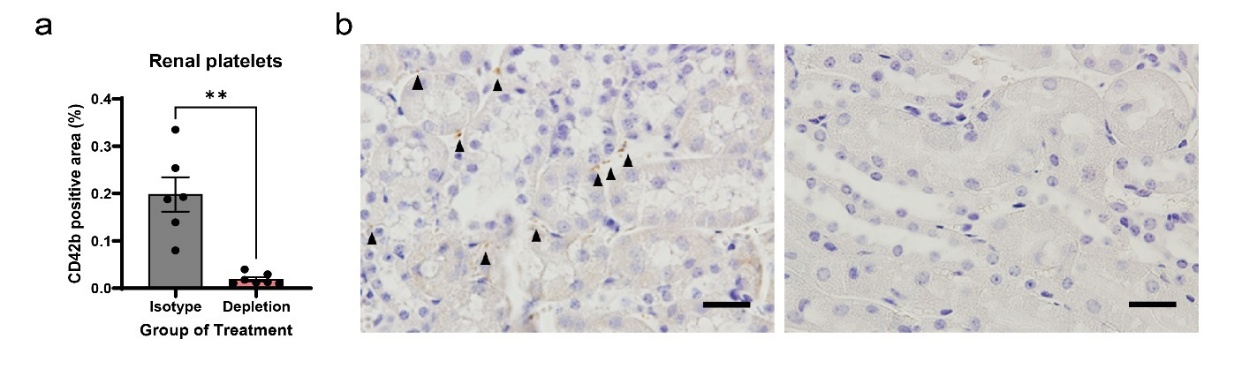


**Figure S2. Renal platelets in the UUO mouse kidney.** (A) The graph shows the percentage of intrarenal platelets determined by immunohistochemistry in the UUO mouse model. (B) Representative images show CD42b staining for platelets in renal tissue sections from the UUO control group (left) and UUO depleted treated group (right). Black arrowheads indicate platelets. Scale bar, 50 µm. Data are presented as mean ±SEM. Statistical analysis was performed using Mann-Whitney U test. ***P*<0.01

**Supplementary Figure S4.**


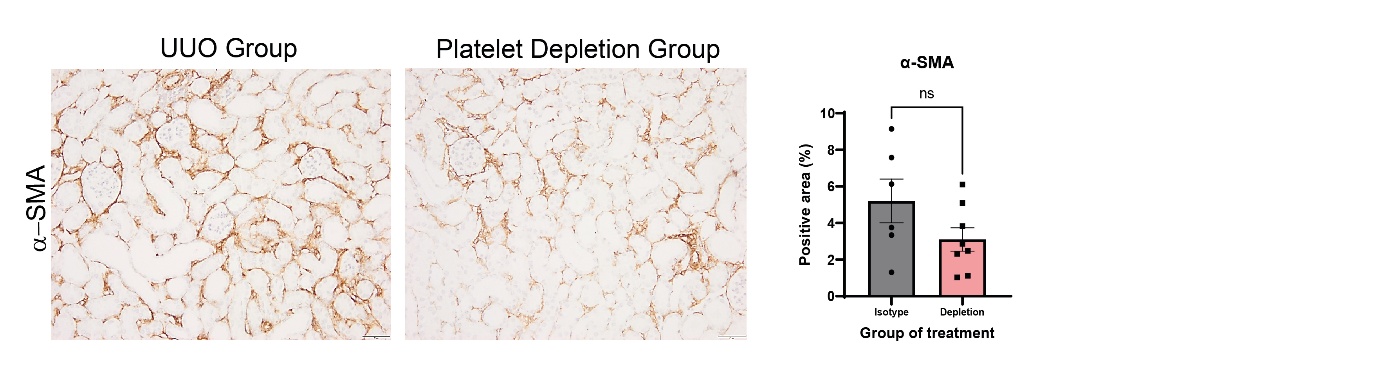


**Figure S4.** **α-SMA staining in UUO mouse kidney.** Representative images show α-SMA staining in renal tissue section from UUO day-3 isotype in control and UUO platelet depleted treated group. The graph shows the percentage of α-SMA-positive staining was quantified in 10 non-overlapping fields using FIJI image analysis software. Scale bar, 50 µm. Data are presented as mean ±SEM. Statistical analysis was performed using the Student’s *t*-test for comparison between two groups.

**Supplementary Figure S5.**


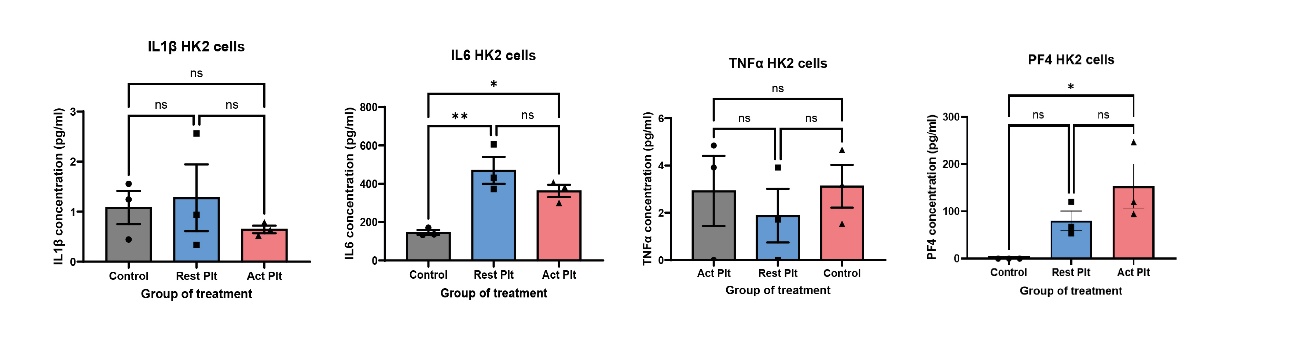


**Figure S5.** **Cytokine and PF4 levels in HK-2 supernatants following platelet stimulation**

Levels of IL-1β, IL-6, TNF-α, and PF4 were measured in culture supernatants from control and platelet-stimulated HK-2 cells. IL-10 was undetectable across all conditions and is not shown. Data are presented as mean ±SEM. Statistical analysis was performed using One-way ANOVA for comparisons involving more than two groups. *P<0.05, **P<0.01
